# Silencing of the foot-and-mouth disease virus internal ribosomal entry site by targeting relatively conserved region among serotypes

**DOI:** 10.1007/s11262-019-01696-6

**Published:** 2019-07-31

**Authors:** Takafumi Matsui, Yoshio Handa, Takehiro Kanda, Kyoko Tsukiyama-Kohara

**Affiliations:** 1 Transboundary Animal Disease Center, Joint Facility of Veterinary Medicine, Kagoshima University, 1-21-24 Korimoto, Kagoshima, Kagoshima 890-0065 Japan; 2 Department of Molecular Virology, Graduate School of Medicine, Kyoto University, 53 Shogoin-kawahara-cho Sakyo-ku, Kyoto, Kyoto 606-8507 Japan; 3 Department of Animal Hygiene, Joint Facility of Veterinary Medicine, Kagoshima University, 1-21-24 Korimoto, Kagoshima, Kagoshima 890-0065 Japan

**Keywords:** Foot-and-mouth disease virus, Internal ribosomal entry site, Short interfering RNA, Short hairpin RNA, Translation

## Abstract

Foot-and-mouth disease (FMD) is a host-restricted disease of cloven-hoofed animals, such as cattle and pigs. There are seven major serotypes of FMD virus that exhibit high antigenic variation, making vaccine strain selection difficult. However, there is an internal ribosomal entry site (IRES) element within the 5′ untranslated region of the FMD virus (FMDV) RNA genome that is relatively conserved among FMDV serotypes and could be used as a pan-serotype target for disease interventions. To determine the potential for targeting the IRES as promising drug target, we designed a short interfering RNA (siRNA) targeting a relatively conserved region in the FMDV-IRES. The siRNA affected FMDV-IRES expression but not the expression of the encephalomyocarditis virus or hepatitis C virus IRES. To evaluate the effects of siRNA-mediated silencing, we established cell lines expressing a bicistronic luciferase reporter plasmid, which contained an FMDV-IRES element between the *Renilla* and firefly luciferase genes. The designed siRNA inhibited FMDV-IRES-mediated translation in a concentration-dependent manner. In order to sustain this inhibitory effect, we designed a short hairpin RNA (shRNA)-expressing lentiviral vector. The results showed that the lenti-shRNA vector significantly suppressed FMDV-IRES activity for up to 2 weeks in cell culture. Thus, our findings in this study provided a basis for the development of effective pan-serotype FMDV inhibitors.

**Electronic supplementary material:**

The online version of this article (10.1007/s11262-019-01696-6) contains supplementary material, which is available to authorized users.

## Introduction

Foot-and-mouth disease (FMD) virus (FMDV; genus *Aphthovirus*, family *Picornaviridae*) is a positive-sense, single-stranded RNA virus that causes FMD, a highly contagious disease of cloven-hoofed animals. FMD is epidemic or sporadic in numerous countries [1], and seven serotypes of FMDV, i.e., O, A, C, Asia 1, SAT1, SAT2, and SAT3 [2], have been identified. FMDV isolates show high levels of genetic diversity [3]. Serotype O is most prevalent, followed by serotype A [4]. The multiple serotypes and variants make disease control difficult; indeed, antigenic differences within a serotype are so great that little or no cross-protection can be achieved between strains of the same serotype [5]. Consistent with these observations, some capsid proteins exhibit sequence variation, with VP1 varying by approximately 30–50% among serotypes [6].

There is an internal ribosomal entry site (IRES) element within the 5′ untranslated region (5′UTR) of the FMDV RNA genome, and this IRES mediates the translation of viral proteins [7, 8]. Other picornaviruses, such as poliovirus (PV) and encephalomyocarditis virus (EMCV), and flaviviruses, such as hepatitis C virus (HCV), possess virus-specific IRES elements within their 5′UTRs [9, 10]. IRES can be classified into five types, designated I (PV), II (FMDV), III (hepatitis A virus), IV (HCV-like), and V (aichivirus-like), based on the higher-order structure [11]. Most eukaryotic mRNAs are translated in a cap-dependent manner, which involves recognition of the 5′ cap structure by the 43S ribosome [12]. Viral mRNA has a short 5′UTR (< 100 nucleotides) and does not contain an initiation AUG, enabling protein synthesis in a cap-dependent manner, similar to most eukaryotic mRNAs [9, 10]. In contrast, IRES-mediated translation is cap-independent [9, 10]. The translation of eukaryotic mRNA is halted by the cleavage of eIF4G with picornavirus protease (e.g., PV 2A^pro^ and FMDV L^pro^), whereas protein synthesis regulated by PV or EMCV-IRES is stimulated [13, 14]. FMDV L^pro^ can enhance translation targeting all picornavirus IRES, even after the inactivation of eIF2 by phosphorylation [15]. Because the IRES region is responsible for translational control functions, its nucleotide sequence is relatively conserved among FMDV serotypes.

In this study, we developed an approach to silence FMDV-IRES by targeting a relatively conserved region among the seven FMDV serotypes in order to gain basic information for the establishment of pan-serotype inhibitors.

## Materials and methods

### Cell culture and plasmids

The human kidney cell line (HEK293) used in this study was obtained and cultured as previously described [16].

The pRF vectors containing FMDV-IRES (serotype C) [17], EMCV-IRES, and HCV-IRES [18] were kind gifts from Dr. Hirasawa and Professor Sung-Key Jang. The pCAGGS-Neo vector was constructed using pCAG Neo (Fujifilm Wako, Tokyo, Japan) and pCAGGS vectors (cat. no. RDB08938; Riken Bank, Ibaraki, Japan). Reporter genes were excised from pCAGGS/FMDV-IRES [16] using the restriction endonucleases *Eco*RV (Toyobo, Osaka, Japan) and *Bam*HI (New England Biolabs, Ipswich, MA, USA). The pCAGGS-Neo/FMDV-IRES vector was generated by inserting a reporter gene into pCAGGS-Neo, which was then treated with *Eco*RV (Toyobo), *Bam*HI (New England Biolabs), and rAPid Alkaline Phosphatase (Roche, Basel, Switzerland) using Mighty Mix (Takara, Shiga, Japan).

The FMDV-IRES short hairpin RNA (shRNA) expression vector was constructed using the pLL3.7 vector (cat. no. 11795; Addgene, Watertown, MA, USA). The shRNA sequence (5′-tACAGGCTAAGGATGCCCTTCAGGTAttcaagagaTACCTGAAGGGCATCCTTAGCCTGTttttttC-3′, where capital letters indicate the target sequence and lower-case letters indicate the loop region) was subcloned under the *U6* promoter.

DNA sequencing was performed by FASMAC Co. (Kanagawa, Japan), and DNA sequence characterization was performed using GENETYX-Mac software (GENETYX Co., Tokyo, Japan) and GENBANK.

### Short interfering RNA (siRNA) transfection

**Fig. 1:**
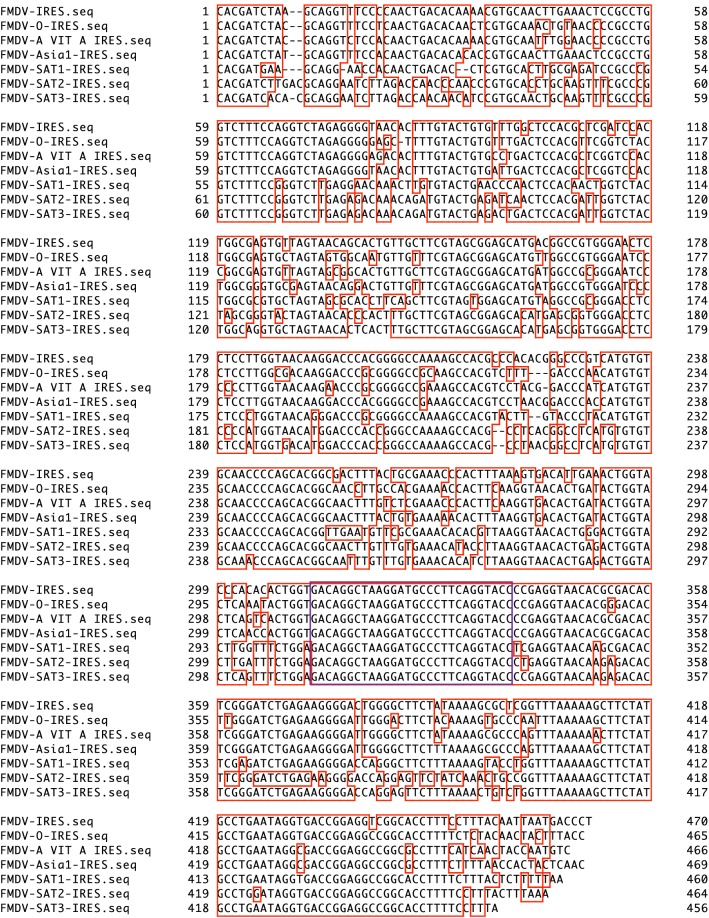
Alignment of FMDV-IRES sequences from seven FMDV serotypes. Alignment of FMDV-IRES sequences from seven serotypes (GENETYX software, multiple alignment). The FMDV-IRES sequences included in this study were classified as serotypes O (GenBank Sequence ID: DQ478936.1), A (GenBank Sequence ID: KY322680.1), Asia 1 (GenBank Sequence ID: AY687333.1), SAT1 (GenBank Sequence ID: MF678823.1), SAT2 (GenBank Sequence ID: KY825724.1), SAT3 (GenBank Sequence ID: KM268901.1), and C (GenBank Sequence ID: AF274010.1). The highly conserved region (nucleotides 312–339) is outlined in purple. The conserved region is outlined in red

siRNA targeting a region of FMDV-IRES conserved among the seven serotypes (FMDV-con siRNA; Fig. 1) was designed as follows using BLOCK-iT RNAi Designer (Thermo Fisher Scientific, Waltham, MA, USA): 5′- ACAGGCUAAGGAUGCCCUUCAGGUA-3′. For control siRNA, ON-target plus siRNA control (Horizon/Dharmacon, Lafayette, CO, USA) was used.

### Northern blot analysis

Total RNA was extracted using ISOGEN (NIPPON GENE Co. Tokyo, Japan) and electrophoresed in formaldehyde agarose (1.2%) gels. Dicistronic mRNA was detected with labeled RNA using a *Pvu*II-digested pRF vector transcribed with T7 RNA polymerase (*Renilla* luciferase region) and detected with digoxigenin (DIF Northern Starter kit; Merk, Darmastadt, Germany).

### Transfection and lentiviral infection

Plasmid transfection was performed using Lipofectamine LTX reagent (Invitrogen, Carlsbad, CA, USA) according to the manufacturer’s specifications after the cells reached 50–70% confluence. For the establishment of cell lines, HEK293 cells were cultured with medium containing G418 (300 μg/mL) after transfection with the pCAGGS-Neo/FMDV-IRES vector. After 3–4 weeks, G418-resistant cells were identified as colonies. siRNA (1, 5, or 10 nM) reverse transfection was performed using Lipofectamine RNAiMAX reagent (Invitrogen) according to the manufacturer’s specifications. Lentiviral packaging was performed using MISSION Lentiviral Packaging Mix (Sigma-Aldrich, St. Louis, MO, USA), and infection with lentivirus was performed according to the manufacturer’s instructions. Titration of lentivirus was performed via detection of green fluorescent protein (GFP) using a fluorescence microscope (Bz-x700; Keyence, Osaka, Japan). Cell viability was measured using WST assays (Dojindo, Kumamoto, Japan) by determining the optical density at 450 nm (OD_450_), according to the manufacturer’s instructions. Luciferase assays were performed using a Dual-Luciferase Reporter Assay System (Promega, Madison, WI, USA). Luminescence was measured with a GloMax 96 Microplate Luminometer (Promega) for 10 s, as previously described [16].

### Statistical analysis

All data are presented as means ± standard deviations from three independent experiments. Statistical analysis was performed using multiple *t* tests corrected for multiple comparisons using the Holm–Sidak method (Graph Pad Prism ver. 8.1.2) to evaluate significant differences. Results with *P* values of less than 0.05 were considered significant.

## Results

### Identification of the conserved region among FMVD-IRES sequences and design of the siRNA sequence

**Fig. 2:**
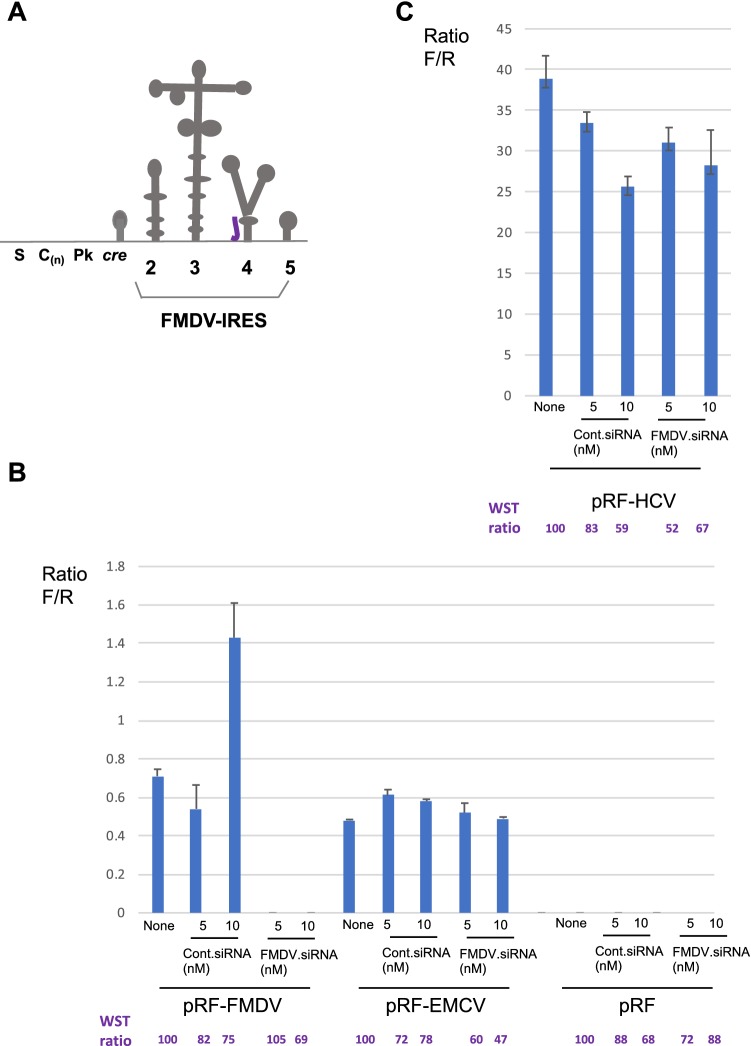
Effects of siRNA on FMDV-IRES, EMCV-IRES, and HCV-IRES. **a** Schematic representation of FMDV-IRES domains 2–5 in the 5′UTR. The purple line represents the position of siRNA. **b** HEK293 cells were cotransfected with FMDV-IRES targeting siRNA and pRF-FMDV, pRF-EMCV, pRF-HCV, and pRF vectors. Firefly and *Renilla* luciferase activities were measured, and IRES activity was calculated as the ratio of firefly luciferase activity to *Renilla* luciferase activity. The ratio (%) of the WST value (OD_450_) relative to the untreated sample is shown under the diagrams

To identify the conserved region among FMDV-IRES sequences, we aligned the 5′UTR sequence (nucleotide numbers 569–1,038 in FMDV serotype C, AF274010.1) with representatives of each of the other six serotypes of FMDV (Fig. 1). As shown in Fig. 1 and Supplementary Fig. 1, the region encompassing nucleotide positions 312–339 in the IRES region was highly conserved among these clones and was chosen for design of siRNA. The designed siRNA was located in the domain 4 region (Fig. 2a) [19], and the efficacy of this siRNA targeting FMDV-IRES was examined by cotransfection with the dicistronic vectors pRF-FMDV-IRES, pRF-EMCV-IRES, and pRF-HCV-IRES (Fig. 2b). Our results showed that FMDV-IRES targeting siRNA could inhibit FMDV-IRES activity but not EMCV-IRES and HCV-IRES activity.

### Establishment of FMDV-IRES-expressing cell lines

In order to evaluate the efficacy of siRNA-mediated FMDV-IRES silencing, we established cell lines containing a bicistronic reporter plasmid [16]. Using *Eco*RV and *Bam*HI, a fragment was excised from the pCAGGS/FMDV-IRES vector [16], which contained an FMDV-IRES element [19] between the *Renilla* and firefly luciferase genes. This fragment was then inserted into the pCAGGS-Neo/MCS vector. The resulting plasmid construct was named pCAGGS-Neo/FMDV-IRES (Supplementary Fig. 2).

**Fig. 3:**
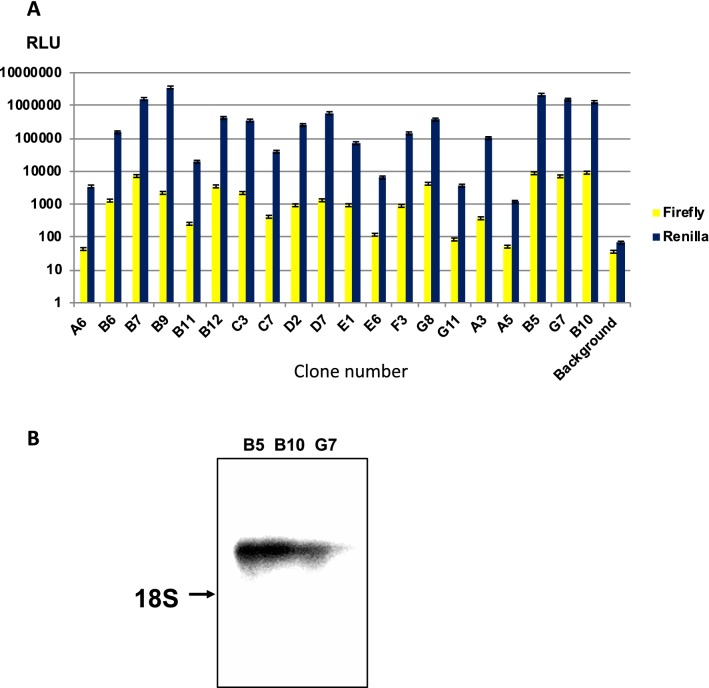
Establishment of cell lines expressing bicistronic luciferase. **a** HEK293 cells were transfected with the pCAGGS-Neo/FMDV-IRES vector. FMDV-IRES-expressing clones were established and verified by measurement of firefly (FMDV-IRES activity) and *Renilla* (cap-dependent translation) luciferase activities. Experiments were performed in triplicate, and error bars indicate standard deviations. **b** Northern blot analysis of dicistronic mRNA in cells. Total RNA (1 μg/lane) extracted from B5, B10, and G7 cells was hybridized with the *Renilla* luciferase RNA probe. The position of 18S rRNA (1.9 kb) is indicated

A previous study indicated that FMDV-IRES activity could be detected in HEK293 cells, similar to other FMDV-susceptible cell lines [16]. Therefore, the above-generated plasmid was then transfected into HEK293 cells and selected by treatment with neomycin. *Renilla* luciferase activity was measured to detect cap-dependent translation, and firefly luciferase activity was measured to detect IRES-mediated translation. We obtained 20 cell lines stably expressing *Renilla* and firefly luciferase, which could then be used for the screening of FMDV-IRES inhibitors (Fig. 3a). We examined the top three clones with the highest FMDV-IRES activities (B5, B10, and G7) and observed similar expression of dicistronic mRNA (3.1 kb, Fig. 3b). From these results, we chose clone B10, which possessed the highest FMDV-IRES activity, for further characterization.

### Effects of siRNA on IRES-mediated translation of FMDV

**Fig. 4:**
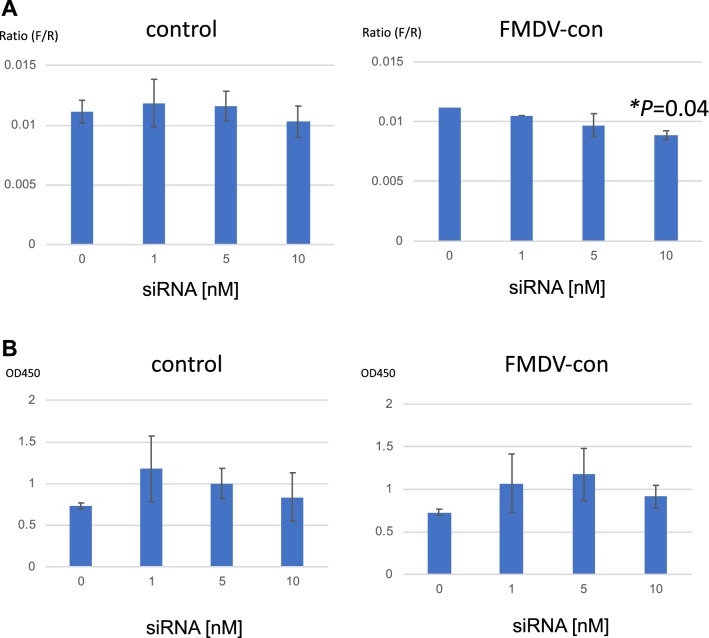
Effects of siRNA on FMDV-IRES activity. **a** siRNA targeting the conserved region of FMDV-IRES was reverse transfected into HEK293 cells (clone B10) using Lipofectamine RNAiMAX and incubated for 48 h. Firefly (FMDV-IRES activity) and *Renilla* (cap-dependent translation) luciferase activities were measured. To evaluate IRES-mediated translational activity, the ratio of IRES-mediated translation to cap-dependent translation was calculated. Experiments were performed in triplicate, and error bars indicate standard deviations. Multiple *t* tests were performed to calculate *p* values (*p < 0.05 compared with siRNA 0 nM) between control cells and cells transfected with FMDV siRNA for each concentration. **b** Cell viability was measured using WST assays by determination of the OD_450_. Experiments were performed in triplicate, and error bars indicate standard deviations

Using FMDV-IRES-expressing cells, we examined the efficacy of the designed siRNA targeting the conserved region of the FMDV-IRES sequence (FMDV-con siRNA, Fig. 4). To evaluate IRES-mediated translational activity, the ratio of IRES-mediated translation to cap-dependent translation was calculated. Treatment with FMDV-con siRNA resulted in inhibition of FMDV-IRES activity in a concentration-dependent manner, whereas control siRNA did not show a significant effect (Fig. 4a). Moreover, treatment with the FMDV-IRES-targeted siRNA did not result in significant cytotoxicity (Fig. 4b).

### Establishment of an shRNA expression vector targeting the FMDV-IRES conserved region

To sustain the effects of silencing, we constructed an shRNA expression vector utilizing a lentiviral expression vector (Supplementary Fig. 3A). We subcloned the shRNA sequence under the control of the *U6* promoter. Transfection with the shRNA expression vector was confirmed with fluorescence microscopy using the *GFP* gene encoded in this vector (Supplementary Fig. 3A, B).

**Fig. 5:**
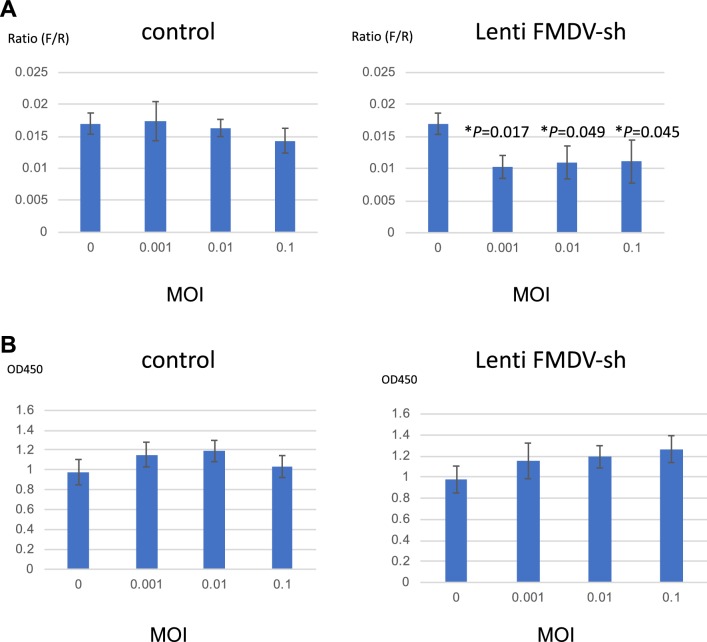
Effects of shRNA on FMDV-IRES activity. **a** The shRNA-expressing lentiviral vector targeting the conserved region of FMDV-IRES (Lenti-FMDV-sh) or control vector (pLL3.7 alone) was used to infect HEK293 cells at various MOIs. After 14 days, firefly (FMDV-IRES activity) and *Renilla* (cap-dependent translation) luciferase activities were measured. To evaluate IRES-mediated translational activity, the ratio of IRES-mediated translation to cap-dependent translation was calculated. Experiments were performed in triplicate, and error bars indicate standard deviations. Multiple *t* tests were performed to calculate *p* values between cells with and without shRNA (**p < 0.05 compared with MOI = 0). **b** Cell viability was measured using WST assays by determining the OD_450_ after 14 days. Experiments were performed in triplicate, and error bars indicate standard deviations

We transduced the resulting Lenti-FMDV-sh vector into cells (clone B10) and measured the IRES activity and cell viability after 14 days (Fig. 5). Transduction with the Lenti-FMDV-sh vector at a multiplicity of infection (MOI) of 0.001–0.1 significantly suppressed FMDV-IRES activity (Fig. 5a). Moreover, transduction with the Lenti-FMDV-sh vector did not significantly affect cell viability (Fig. 5b).

## Discussion

In this study, we evaluated the silencing effects of siRNA and shRNA targeting a conserved region of the FMDV-IRES among the seven FMDV serotypes. The lenti-shRNA expression vector generated shRNA under control of the *U6* promoter persistently [20], enabling observation of its suppressive effects on FMDV-IRES-mediated translational activity in HEK293 cells after 14 days without significant cytotoxicity. These features make this construct promising in terms of solving several current challenges with FMDV vaccination. Moreover, this lenti-shRNA expression vector could be applicable to transduction in embryos for construction of transgenic animals [21]. For establishment of therapeutic vectors in vivo in future studies, it will be necessary to use vectors other than lentiviruses, e.g., adenovirus vectors [22, 23] and adeno-associated virus vectors [24].

FMDV populations show high levels of genetic diversity, mainly because of the lack of RNA polymerase proofreading ability. This diversity makes disease control using vaccines and laboratory diagnosis difficult [3]. For example, because of antigenic diversity between serotypes and genotypes, vaccination with another serotype or genotype of the same serotype may fail to control disease [25]. In addition, new variant viruses generated after vaccination through escape mutation can cause vaccine failure [26]. However, for the IRES element, the nucleotide sequence is relatively more conserved than that of viral structural proteins. Because IRES function is supported by higher-order RNA structures, including stem-loop structures, disruption of these structures decreases IRES activity. Thus, IRES mutants do not replicate efficiently, selecting for relative conservation of the IRES region [27]. Moreover, the IRES conserved region could be a target for prevention of FMDV infection [28, 29]. This makes the IRES region a suitable target for pan-serotype antiviral drugs for FMDV. However, there is also a risk for generating resistant escape mutant viruses [30], and this should be evaluated in future studies.

The efficacies of vaccines and drugs can be influenced by the host animal species. For example, levels of FMDV replication are significantly higher in pigs than in other animals [31], which may be related to the influence of host factors on RNA replication. However, for IRES-mediated translation, activity is not significantly influenced by the cell line origin [16], and host translation factors (e.g., eIF4E, eIF2, and eIF3) are highly conserved among animal species. Therefore, FMDV-IRES shRNA is expected to be effective in all animal species.

FMDV is a highly contagious agent that can be studied only in special facilities with highly regulated biosecurity protocols [32]. The FMDV-IRES-expressing cells established in this study could enable the screening of new inhibitors of FMDV replication in laboratories with less stringent security clearance.

In summary, the IRES-mediated translational activity of FMDV may be a suitable target for the development of pan-serotype antiviral drugs because of the relatively high sequence conservation of IRES among FMDV serotypes. The FMDV-IRES shRNA-expressing vector and FMDV-IRES-expressing cells established in this study provide new tools for the screening of anti-FMDV drugs. Future studies to evaluate antiviral effects and improve the shRNA delivery vector system are required for establishment of anti-FMDV drugs.

## Electronic supplementary material

Below is the link to the electronic supplementary material.

Supplementary material 1 (PDF 201 kb) Supplementary Fig. 1 Alignment of FMDV strain sequences with 100% homology to the siRNA con sequence by NCBI Blast search. Comparisons with 100 strain sequences are shown

Supplementary material 2 (PDF 13719 kb) Supplementary Fig. 2 Structure of the bicistronic luciferase reporter construct A bicistronic reporter construct was designed to contain the FMDV-IRES element located between the *Renilla* and firefly luciferase genes. The bicistronic reporter gene was cloned using the restriction enzymes *Eco*RV and *Bam*HI and was ligated into the pCAGGS-Neo/MCS vector digested with *Eco*RV and *Bam*HI. Supplementary Fig. 3 Construction of the FMDV-IRES-targeting shRNA expression vector. (A) The FMDV-IRES-targeting shRNA expression vector was constructed using the pLL3.7 vector. CMV: human cytomegalovirus; EGFP: enhanced green fluorescent protein; 5′LTR: truncated 5′ long terminal repeat from HIV-1; RRE: Rev response element of HIV-1; WPRE: woodchuck hepatitis virus posttranscriptional regulatory element. (B) Transfection of cells with shRNA expression vector was observed by fluorescence microscopy (a–f). Fluorescent images (a, g: 40 × ; d: 200 ×), translucent images (b, h: 40 × ; e: 200 ×), and merged images (c, i: 40 × ; f: 200 ×) are shown. Mock transfection controls (g–i) are also shown
