# Supplementary material for: Silencing of the foot-and-mouth disease virus internal ribosomal entry site by targeting relatively conserved region among serotypes
Source: Virus Genes. 2019 Jul 31;55(6):786–94. doi: 10.1007/s11262-019-01696-6 (PMC6831537; doi:10.1007/s11262-019-01696-6)
Supplement: Supplementary file 1 — Supplementary material 1 (PDF 201 kb) Supplementary Fig. 1 Alignment of FMDV strain sequences with 100% homology to the siRNA con sequence by NCBI Blast search. Comparisons with 100 strain sequences are shown [file 11262_2019_1696_MOESM1_ESM.pdf]

Descriptions

Sequences producing significant alignments:

Description	Max Score	Total Score	Query Cover	E value	Per. Ident	Accession
Foot-and-mouth disease virus - type O O/JPN/2010-1/14c (GNa) genomic RNA, nearly complete genome	50.1	50.1	100%	0.001	100.00%	LC485149.1
Foot-and-mouth disease virus - type O O/JPN/2010-1/14c (GSe) genomic RNA, nearly complete genome	50.1	50.1	100%	0.001	100.00%	LC485148.1
Foot-and-mouth disease virus - type O O/JPN/2010-1/14c (SV2_d1) genomic RNA, nearly complete genome	50.1	50.1	100%	0.001	100.00%	LC485147.1
Foot-and-mouth disease virus - type O O/JPN/2010-1/14c (SV_d1) genomic RNA, nearly complete genome	50.1	50.1	100%	0.001	100.00%	LC485146.1
Foot-and-mouth disease virus - type O O/JPN/2010-1/14c (SV_d6) genomic RNA, nearly complete genome	50.1	50.1	100%	0.001	100.00%	LC485145.1
Foot-and-mouth disease virus - type O O/JPN/2010-1/14c (SV_d3) genomic RNA, nearly complete genome	50.1	50.1	100%	0.001	100.00%	LC485144.1
Foot-and-mouth disease virus - type O O/JPN/2010-1/14c (CV2_d1) genomic RNA, nearly complete genome	50.1	50.1	100%	0.001	100.00%	LC485143.1
Foot-and-mouth disease virus - type O O/JPN/2010-1/14c (CV_d1) genomic RNA, nearly complete genome	50.1	50.1	100%	0.001	100.00%	LC485142.1
Foot-and-mouth disease virus - type O O/MYA/Yan/5/2016 gene for polyprotein, complete cds	50.1	50.1	100%	0.001	100.00%	LC438823.1
Foot-and-mouth disease virus - type O O/MYA/Yan/3/2016 gene for polyprotein, complete cds	50.1	50.1	100%	0.001	100.00%	LC438822.1
Foot-and-mouth disease virus - type O strain O/VN1/2014, complete genome	50.1	50.1	100%	0.001	100.00%	MH845413.2
Foot-and-mouth disease virus strain IRFP1 5' UTR	50.1	50.1	100%	0.001	100.00%	MG678804.1
Foot-and-mouth disease virus - type O strain O/VIT/8338/2017 polyprotein gene, complete cds	50.1	50.1	100%	0.001	100.00%	MH891503.1
Foot-and-mouth disease virus - type O isolate PAK/14/2017, complete genome	50.1	50.1	100%	0.001	100.00%	MH784405.1
Foot-and-mouth disease virus - type O isolate PAK/4/2017, complete genome	50.1	50.1	100%	0.001	100.00%	MH784404.1
Foot-and-mouth disease virus - type O isolate PAK/10/2016, complete genome	50.1	50.1	100%	0.001	100.00%	MH784403.1
Foot-and-mouth disease virus - type O isolate VIT/20/2016, partial genome	50.1	50.1	100%	0.001	100.00%	MG983741.1
Foot-and-mouth disease virus - type O isolate VIT/8/2015, partial genome	50.1	50.1	100%	0.001	100.00%	MG983740.1
Foot-and-mouth disease virus - type O isolate UAE/3/2016, partial genome	50.1	50.1	100%	0.001	100.00%	MG983739.1
Foot-and-mouth disease virus - type O isolate UAE/1/2016, partial genome	50.1	50.1	100%	0.001	100.00%	MG983738.1
Foot-and-mouth disease virus - type O isolate UAE/2/2014, partial genome	50.1	50.1	100%	0.001	100.00%	MG983737.1
Foot-and-mouth disease virus - type O isolate UAE/1/2014, partial genome	50.1	50.1	100%	0.001	100.00%	MG983736.1

Foot-and-mouth disease virus - type O isolate TUN/1/2014, partial genome	50.1	50.1	100%	0.001	100.00%	MG983735.1
Foot-and-mouth disease virus - type O isolate SRL/30/2014, partial genome	50.1	50.1	100%	0.001	100.00%	MG983734.1
Foot-and-mouth disease virus - type O isolate SRL/28/2014, partial genome	50.1	50.1	100%	0.001	100.00%	MG983733.1
Foot-and-mouth disease virus - type O isolate SRL/1/2014, partial genome	50.1	50.1	100%	0.001	100.00%	MG983732.1
Foot-and-mouth disease virus - type O isolate SAU/2/2016, complete genome	50.1	50.1	100%	0.001	100.00%	MG983729.1
Foot-and-mouth disease virus - type O isolate SAU/1/2016, partial genome	50.1	50.1	100%	0.001	100.00%	MG983728.1
Foot-and-mouth disease virus - type O isolate SAU/20/2015, partial genome	50.1	50.1	100%	0.001	100.00%	MG983727.1
Foot-and-mouth disease virus - type O isolate SAU/4/2014, partial genome	50.1	50.1	100%	0.001	100.00%	MG983726.1
Foot-and-mouth disease virus - type O isolate SAU/1/2014, partial genome	50.1	50.1	100%	0.001	100.00%	MG983725.1
Foot-and-mouth disease virus - type O isolate SAU/7/2013, partial genome	50.1	50.1	100%	0.001	100.00%	MG983724.1
Foot-and-mouth disease virus - type O isolate SAU/6/2013, partial genome	50.1	50.1	100%	0.001	100.00%	MG983723.1
Foot-and-mouth disease virus - type O isolate SAU/4/2013, partial genome	50.1	50.1	100%	0.001	100.00%	MG983722.1
Foot-and-mouth disease virus - type O isolate SAU/1/2013, partial genome	50.1	50.1	100%	0.001	100.00%	MG983721.1
Foot-and-mouth disease virus - type O isolate NEP/21/2015, complete genome	50.1	50.1	100%	0.001	100.00%	MG983719.1
Foot-and-mouth disease virus - type O isolate NEP/10/2015, partial genome	50.1	50.1	100%	0.001	100.00%	MG983718.1
Foot-and-mouth disease virus - type O isolate NEP/3/2015, partial genome	50.1	50.1	100%	0.001	100.00%	MG983717.1
Foot-and-mouth disease virus - type O isolate NEP/6/2014, partial genome	50.1	50.1	100%	0.001	100.00%	MG983716.1
Foot-and-mouth disease virus - type O isolate NEP/1/2014, partial genome	50.1	50.1	100%	0.001	100.00%	MG983715.1
Foot-and-mouth disease virus - type O isolate NEP/17/2013, partial genome	50.1	50.1	100%	0.001	100.00%	MG983713.1
Foot-and-mouth disease virus - type O isolate NEP/16/2013, partial genome	50.1	50.1	100%	0.001	100.00%	MG983712.1
Foot-and-mouth disease virus - type O isolate NEP/21/2012, partial genome	50.1	50.1	100%	0.001	100.00%	MG983710.1
Foot-and-mouth disease virus - type O isolate NEP/13/2012, partial genome	50.1	50.1	100%	0.001	100.00%	MG983709.1
Foot-and-mouth disease virus - type O isolate NEP/10/2012, partial genome	50.1	50.1	100%	0.001	100.00%	MG983708.1
Foot-and-mouth disease virus - type O isolate NEP/6/2012, partial genome	50.1	50.1	100%	0.001	100.00%	MG983707.1
Foot-and-mouth disease virus - type O isolate NEP/9/2010, partial genome	50.1	50.1	100%	0.001	100.00%	MG983706.1
Foot-and-mouth disease virus - type O isolate NEP/7/2010, partial genome	50.1	50.1	100%	0.001	100.00%	MG983705.1
Foot-and-mouth disease virus - type O isolate NEP/5/2008, partial genome	50.1	50.1	100%	0.001	100.00%	MG983704.1
Foot-and-mouth disease virus - type O isolate MUR/23/2016, partial genome	50.1	50.1	100%	0.001	100.00%	MG983702.1
Foot-and-mouth disease virus - type O isolate MUR/21/2016, partial genome	50.1	50.1	100%	0.001	100.00%	MG983701.1
Foot-and-mouth disease virus - type O isolate MUR/19/2016, partial genome	50.1	50.1	100%	0.001	100.00%	MG983700.1
Foot-and-mouth disease virus - type O	50.1	50.1	100%	0.001	100.00%	MG983699.1

isolate MUR/9/2016, partial genome						
Foot-and-mouth disease virus - type O isolate MUR/5/2016, partial genome	50.1	50.1	100%	0.001	100.00%	MG983698.1
Foot-and-mouth disease virus - type O isolate LIB/22/2013, partial genome	50.1	50.1	100%	0.001	100.00%	MG983697.1
Foot-and-mouth disease virus - type O isolate LIB/17/2013, partial genome	50.1	50.1	100%	0.001	100.00%	MG983696.1
Foot-and-mouth disease virus - type O isolate LIB/7/2013, complete genome	50.1	50.1	100%	0.001	100.00%	MG983695.1
Foot-and-mouth disease virus - type O isolate LIB/1/2013, partial genome	50.1	50.1	100%	0.001	100.00%	MG983694.1
Foot-and-mouth disease virus - type O isolate LAO/2/2015, partial genome	50.1	50.1	100%	0.001	100.00%	MG983693.1
Foot-and-mouth disease virus - type O isolate IRN/72/2009, partial genome	50.1	50.1	100%	0.001	100.00%	MG983692.1
Foot-and-mouth disease virus - type O isolate BHU/3/2016, partial genome	50.1	50.1	100%	0.001	100.00%	MG983690.1
Foot-and-mouth disease virus - type O isolate BHU/12/2012, partial genome	50.1	50.1	100%	0.001	100.00%	MG983689.1
Foot-and-mouth disease virus - type O isolate BHU/40/2009, partial genome	50.1	50.1	100%	0.001	100.00%	MG983688.1
Foot-and-mouth disease virus - type O isolate BHU/2/2009, partial genome	50.1	50.1	100%	0.001	100.00%	MG983687.1
Foot-and-mouth disease virus - type O isolate BAR/15/2015, partial genome	50.1	50.1	100%	0.001	100.00%	MG983686.1
Foot-and-mouth disease virus - type O isolate BAR/2/2015, partial genome	50.1	50.1	100%	0.001	100.00%	MG983685.1
Foot-and-mouth disease virus - type O isolate BAN/1/2009, partial genome	50.1	50.1	100%	0.001	100.00%	MG983684.1
Foot-and-mouth disease virus - type O isolate ALG/1/2014, partial genome	50.1	50.1	100%	0.001	100.00%	MG983683.1
Foot-and-mouth disease virus - type C isolate C1 OBERBAYERN, complete genome	50.1	50.1	100%	0.001	100.00%	MG372732.1
Foot-and-mouth disease virus - type Asia 1 isolate TBD, complete genome	50.1	50.1	100%	0.001	100.00%	MG372731.1
Foot-and-mouth disease virus - type O isolate O1 S. Korea 2000, complete genome	50.1	50.1	100%	0.001	100.00%	MG372730.1
Foot-and-mouth disease virus - type C isolate C3 INDAIAL, complete genome	50.1	50.1	100%	0.001	100.00%	MG372729.1
Foot-and-mouth disease virus - type O isolate O PENGHU, complete genome	50.1	50.1	100%	0.001	100.00%	MG372728.1
Foot-and-mouth disease virus - type SAT 3 isolate SAT 3/2 S. Africa, complete genome	50.1	50.1	100%	0.001	100.00%	MG372727.1
Foot-and-mouth disease virus - type Asia 1 isolate IND281/1994, complete genome	50.1	50.1	100%	0.001	100.00%	MF372126.1
Foot-and-mouth disease virus - type A isolate BAN/CH/Sa-304/2016, complete genome	50.1	50.1	100%	0.001	100.00%	MK088171.1
Foot-and-mouth disease virus - type O isolate BAN/BO/Na-161/2013, complete genome	50.1	50.1	100%	0.001	100.00%	MK071699.1
Foot-and-mouth disease virus - type A isolate VIT/42/2013, complete genome	50.1	50.1	100%	0.001	100.00%	KY322680.1
Foot-and-mouth disease virus - type A isolate TAI/4/2014, complete genome	50.1	50.1	100%	0.001	100.00%	KY322679.1
Foot-and-mouth disease virus - type A isolate MAY/23/2013, complete genome	50.1	50.1	100%	0.001	100.00%	KY322678.1
Foot-and-mouth disease virus - type A isolate MAY/20/2013, complete genome	50.1	50.1	100%	0.001	100.00%	KY322677.1

Foot-and-mouth disease virus - type A isolate MAY/12/2013, complete genome	50.1	50.1	100%	0.001	100.00%	KY322676.1
Foot-and-mouth disease virus - type A isolate LAO/3/2014, complete genome	50.1	50.1	100%	0.001	100.00%	KY322675.1
Foot-and-mouth disease virus - type O isolate SKR/6/2014, complete genome	50.1	50.1	100%	0.001	100.00%	KY322674.1
Foot-and-mouth disease virus - type O isolate MAY/8/2014, complete genome	50.1	50.1	100%	0.001	100.00%	KY322673.1
Foot-and-mouth disease virus - type O isolate MAY/3/2014, complete genome	50.1	50.1	100%	0.001	100.00%	KY322672.1
Foot-and-mouth disease virus - type O isolate MAY/2/2014, complete genome	50.1	50.1	100%	0.001	100.00%	KY322671.1
Foot-and-mouth disease virus - type O isolate LAO/1/2013, complete genome	50.1	50.1	100%	0.001	100.00%	KY322670.1
Synthetic construct NP and VP35 genes, complete cds	50.1	50.1	100%	0.001	100.00%	MF801600.1
Foot-and-mouth disease virus - type SAT 1 isolate SAT1/NIG/4/15, complete genome	50.1	50.1	100%	0.001	100.00%	MF678826.1
Foot-and-mouth disease virus - type SAT 1 isolate SAT1/NIG/3/15, complete genome	50.1	50.1	100%	0.001	100.00%	MF678825.1
Foot-and-mouth disease virus - type SAT 1 isolate SAT1/NIG/2/15, complete genome	50.1	50.1	100%	0.001	100.00%	MF678824.1
Foot-and-mouth disease virus - type SAT 1 isolate SAT1/NIG/1/15, complete genome	50.1	50.1	100%	0.001	100.00%	MF678823.1
Foot-and-mouth disease virus - type Asia 1 strain Asia1-9/Shamir/ISR/89 polyprotein gene, partial cds	50.1	50.1	100%	0.001	100.00%	MF063056.1
Foot-and-mouth disease virus - type Asia 1 strain Asia1-3/Shamir/ISR/89 polyprotein gene, partial cds	50.1	50.1	100%	0.001	100.00%	MF063054.1
Foot-and-mouth disease virus - type Asia 1 strain Asia1/Shamir/ISR/89 polyprotein gene, complete cds	50.1	50.1	100%	0.001	100.00%	MF063053.1
Foot-and-mouth disease virus isolate Diwanyia No.4 5' UTR	50.1	50.1	100%	0.001	100.00%	MF116381.1
Foot-and-mouth disease virus isolate Diwanyia No.2 5' UTR	50.1	50.1	100%	0.001	100.00%	MF116379.1
Foot-and-mouth disease virus isolate Diwanyia No.1 5' UTR	50.1	50.1	100%	0.001	100.00%	MF116378.1
Foot-and-mouth disease virus - type A isolate A01NL, complete genome	50.1	50.1	100%	0.001	100.00%	KY404935.1

Alignments

Foot-and-mouth disease virus - type O O/JPN/2010-1/14c (GNa) genomic RNA, nearly complete genome

Sequence ID: **LC485149.1** Length: 7718 Number of Matches: 1

Range 1: 525 to 549

Score	Expect	Identities	Gaps	Strand	Frame
50.1 bits(25)	0.001()	25/25(100%)	0/25(0%)	Plus/Plus	

Features:

```
Query  1    ACAGGCTAAGGATGCCCTTCAGGTA  25
            |||||||||||||||||||||||||
Sbjct  525  ACAGGCTAAGGATGCCCTTCAGGTA  549
```

Foot-and-mouth disease virus - type O O/JPN/2010-1/14c (GSe) genomic RNA, nearly complete genome

Sequence ID: **LC485148.1** Length: 7718 Number of Matches: 1

Range 1: 525 to 549

Score	Expect	Identities	Gaps	Strand	Frame

Score	Expect	Identities	Gaps	Strand	Frame
50.1 bits(25)	0.001()	25/25(100%)	0/25(0%)	Plus/Plus	

Features:

```
Query  1    ACAGGCTAAGGATGCCCTTCAGGTA  25
            |||||||||||||||||||||||||
Sbjct  525  ACAGGCTAAGGATGCCCTTCAGGTA  549
```

Foot-and-mouth disease virus - type O O/JPN/2010-1/14c (SV2_d1) genomic RNA, nearly complete genome
Sequence ID: **LC485147.1** Length: 7718 Number of Matches: 1
Range 1: 525 to 549

Score	Expect	Identities	Gaps	Strand	Frame
50.1 bits(25)	0.001()	25/25(100%)	0/25(0%)	Plus/Plus	

Features:

```
Query  1    ACAGGCTAAGGATGCCCTTCAGGTA  25
            |||||||||||||||||||||||||
Sbjct  525  ACAGGCTAAGGATGCCCTTCAGGTA  549
```

Foot-and-mouth disease virus - type O O/JPN/2010-1/14c (SV_d1) genomic RNA, nearly complete genome
Sequence ID: **LC485146.1** Length: 7715 Number of Matches: 1
Range 1: 525 to 549

Score	Expect	Identities	Gaps	Strand	Frame
50.1 bits(25)	0.001()	25/25(100%)	0/25(0%)	Plus/Plus	

Features:

```
Query  1    ACAGGCTAAGGATGCCCTTCAGGTA  25
            |||||||||||||||||||||||||
Sbjct  525  ACAGGCTAAGGATGCCCTTCAGGTA  549
```

Foot-and-mouth disease virus - type O O/JPN/2010-1/14c (SV_d6) genomic RNA, nearly complete genome
Sequence ID: **LC485145.1** Length: 7719 Number of Matches: 1
Range 1: 525 to 549

Score	Expect	Identities	Gaps	Strand	Frame
50.1 bits(25)	0.001()	25/25(100%)	0/25(0%)	Plus/Plus	

Features:

```
Query  1    ACAGGCTAAGGATGCCCTTCAGGTA  25
            |||||||||||||||||||||||||
Sbjct  525  ACAGGCTAAGGATGCCCTTCAGGTA  549
```

Foot-and-mouth disease virus - type O O/JPN/2010-1/14c (SV_d3) genomic RNA, nearly complete genome
Sequence ID: **LC485144.1** Length: 7718 Number of Matches: 1
Range 1: 525 to 549

Score	Expect	Identities	Gaps	Strand	Frame
50.1 bits(25)	0.001()	25/25(100%)	0/25(0%)	Plus/Plus	

Features:

```
Query  1    ACAGGCTAAGGATGCCCTTCAGGTA  25
            |||||||||||||||||||||||||
Sbjct  525  ACAGGCTAAGGATGCCCTTCAGGTA  549
```

Foot-and-mouth disease virus - type O O/JPN/2010-1/14c (CV2_d1) genomic RNA, nearly complete genome
Sequence ID: **LC485143.1** Length: 7715 Number of Matches: 1
Range 1: 525 to 549

Score	Expect	Identities	Gaps	Strand	Frame
50.1 bits(25)	0.001()	25/25(100%)	0/25(0%)	Plus/Plus	

Features:

```
Query  1    ACAGGCTAAGGATGCCCTTCAGGTA  25
```

```
                ||||||||||||||||||||||||||
Sbjct  525  ACAGGCTAAGGATGCCCTTCAGGTA  549
```

Foot-and-mouth disease virus - type O O/JPN/2010-1/14c (CV_d1) genomic RNA, nearly complete genome
Sequence ID: **LC485142.1** Length: 7719 Number of Matches: 1
Range 1: 525 to 549

Score	Expect	Identities	Gaps	Strand	Frame
50.1 bits(25)	0.001()	25/25(100%)	0/25(0%)	Plus/Plus	

Features:

```
Query  1    ACAGGCTAAGGATGCCCTTCAGGTA  25
            |||||||||||||||||||||||||
Sbjct  525  ACAGGCTAAGGATGCCCTTCAGGTA  549
```

Foot-and-mouth disease virus - type O O/MYA/Yan/5/2016 gene for polyprotein, complete cds
Sequence ID: **LC438823.1** Length: 7733 Number of Matches: 1
Range 1: 540 to 564

Score	Expect	Identities	Gaps	Strand	Frame
50.1 bits(25)	0.001()	25/25(100%)	0/25(0%)	Plus/Plus	

Features:

```
Query  1    ACAGGCTAAGGATGCCCTTCAGGTA  25
            |||||||||||||||||||||||||
Sbjct  540  ACAGGCTAAGGATGCCCTTCAGGTA  564
```

Foot-and-mouth disease virus - type O O/MYA/Yan/3/2016 gene for polyprotein, complete cds
Sequence ID: **LC438822.1** Length: 7692 Number of Matches: 1
Range 1: 497 to 521

Score	Expect	Identities	Gaps	Strand	Frame
50.1 bits(25)	0.001()	25/25(100%)	0/25(0%)	Plus/Plus	

Features:

```
Query  1    ACAGGCTAAGGATGCCCTTCAGGTA  25
            |||||||||||||||||||||||||
Sbjct  497  ACAGGCTAAGGATGCCCTTCAGGTA  521
```

Foot-and-mouth disease virus - type O strain O/VN1/2014, complete genome
Sequence ID: **MH845413.2** Length: 8131 Number of Matches: 1
Range 1: 854 to 878

Score	Expect	Identities	Gaps	Strand	Frame
50.1 bits(25)	0.001()	25/25(100%)	0/25(0%)	Plus/Plus	

Features:

```
Query  1    ACAGGCTAAGGATGCCCTTCAGGTA  25
            |||||||||||||||||||||||||
Sbjct  854  ACAGGCTAAGGATGCCCTTCAGGTA  878
```

Foot-and-mouth disease virus strain IRFP1 5' UTR
Sequence ID: **MG678804.1** Length: 291 Number of Matches: 1
Range 1: 222 to 246

Score	Expect	Identities	Gaps	Strand	Frame
50.1 bits(25)	0.001()	25/25(100%)	0/25(0%)	Plus/Plus	

Features:

```
Query  1    ACAGGCTAAGGATGCCCTTCAGGTA  25
            |||||||||||||||||||||||||
Sbjct  222  ACAGGCTAAGGATGCCCTTCAGGTA  246
```

Foot-and-mouth disease virus - type O strain O/VIT/8338/2017 polyprotein gene, complete cds
Sequence ID: **MH891503.1** Length: 7787 Number of Matches: 1
Range 1: 535 to 559

Score	Expect	Identities	Gaps	Strand	Frame
50.1 bits(25)	0.001()	25/25(100%)	0/25(0%)	Plus/Plus	

Features:

```
Query  1    ACAGGCTAAGGATGCCCTTCAGGTA  25
            |||||||||||||||||||||||||
Sbjct  535  ACAGGCTAAGGATGCCCTTCAGGTA  559
```

Foot-and-mouth disease virus - type O isolate PAK/14/2017, complete genome
Sequence ID: **MH784405.1** Length: 8197 Number of Matches: 1
Range 1: 937 to 961

Score	Expect	Identities	Gaps	Strand	Frame
50.1 bits(25)	0.001()	25/25(100%)	0/25(0%)	Plus/Plus	

Features:

```
Query  1    ACAGGCTAAGGATGCCCTTCAGGTA  25
            |||||||||||||||||||||||||
Sbjct  937  ACAGGCTAAGGATGCCCTTCAGGTA  961
```

Foot-and-mouth disease virus - type O isolate PAK/4/2017, complete genome
Sequence ID: **MH784404.1** Length: 8194 Number of Matches: 1
Range 1: 936 to 960

Score	Expect	Identities	Gaps	Strand	Frame
50.1 bits(25)	0.001()	25/25(100%)	0/25(0%)	Plus/Plus	

Features:

```
Query  1    ACAGGCTAAGGATGCCCTTCAGGTA  25
            |||||||||||||||||||||||||
Sbjct  936  ACAGGCTAAGGATGCCCTTCAGGTA  960
```

Foot-and-mouth disease virus - type O isolate PAK/10/2016, complete genome
Sequence ID: **MH784403.1** Length: 8193 Number of Matches: 1
Range 1: 936 to 960

Score	Expect	Identities	Gaps	Strand	Frame
50.1 bits(25)	0.001()	25/25(100%)	0/25(0%)	Plus/Plus	

Features:

```
Query  1    ACAGGCTAAGGATGCCCTTCAGGTA  25
            |||||||||||||||||||||||||
Sbjct  936  ACAGGCTAAGGATGCCCTTCAGGTA  960
```

Foot-and-mouth disease virus - type O isolate VIT/20/2016, partial genome
Sequence ID: **MG983741.1** Length: 8206 Number of Matches: 1
Range 1: 932 to 956

Score	Expect	Identities	Gaps	Strand	Frame
50.1 bits(25)	0.001()	25/25(100%)	0/25(0%)	Plus/Plus	

Features:

```
Query  1    ACAGGCTAAGGATGCCCTTCAGGTA  25
            |||||||||||||||||||||||||
Sbjct  932  ACAGGCTAAGGATGCCCTTCAGGTA  956
```

Foot-and-mouth disease virus - type O isolate VIT/8/2015, partial genome
Sequence ID: **MG983740.1** Length: 8197 Number of Matches: 1
Range 1: 934 to 958

Score	Expect	Identities	Gaps	Strand	Frame
50.1 bits(25)	0.001()	25/25(100%)	0/25(0%)	Plus/Plus	

Features:

```
Query  1    ACAGGCTAAGGATGCCCTTCAGGTA  25
            |||||||||||||||||||||||||
Sbjct  934  ACAGGCTAAGGATGCCCTTCAGGTA  958
```

Foot-and-mouth disease virus - type O isolate UAE/3/2016, partial genome
Sequence ID: **MG983739.1** Length: 8162 Number of Matches: 1
Range 1: 912 to 936

Score	Expect	Identities	Gaps	Strand	Frame
50.1 bits(25)	0.001()	25/25(100%)	0/25(0%)	Plus/Plus	

Features:

```
Query  1    ACAGGCTAAGGATGCCCTTCAGGTA  25
            |||||||||||||||||||||||||
Sbjct  912  ACAGGCTAAGGATGCCCTTCAGGTA  936
```

Foot-and-mouth disease virus - type O isolate UAE/1/2016, partial genome
Sequence ID: **MG983738.1** Length: 8188 Number of Matches: 1
Range 1: 933 to 957

Score	Expect	Identities	Gaps	Strand	Frame
50.1 bits(25)	0.001()	25/25(100%)	0/25(0%)	Plus/Plus	

Features:

```
Query  1    ACAGGCTAAGGATGCCCTTCAGGTA  25
            |||||||||||||||||||||||||
Sbjct  933  ACAGGCTAAGGATGCCCTTCAGGTA  957
```

Foot-and-mouth disease virus - type O isolate UAE/2/2014, partial genome
Sequence ID: **MG983737.1** Length: 8182 Number of Matches: 1
Range 1: 925 to 949

Score	Expect	Identities	Gaps	Strand	Frame
50.1 bits(25)	0.001()	25/25(100%)	0/25(0%)	Plus/Plus	

Features:

```
Query  1    ACAGGCTAAGGATGCCCTTCAGGTA  25
            |||||||||||||||||||||||||
Sbjct  925  ACAGGCTAAGGATGCCCTTCAGGTA  949
```

Foot-and-mouth disease virus - type O isolate UAE/1/2014, partial genome
Sequence ID: **MG983736.1** Length: 8188 Number of Matches: 1
Range 1: 930 to 954

Score	Expect	Identities	Gaps	Strand	Frame
50.1 bits(25)	0.001()	25/25(100%)	0/25(0%)	Plus/Plus	

Features:

```
Query  1    ACAGGCTAAGGATGCCCTTCAGGTA  25
            |||||||||||||||||||||||||
Sbjct  930  ACAGGCTAAGGATGCCCTTCAGGTA  954
```

Foot-and-mouth disease virus - type O isolate TUN/1/2014, partial genome
Sequence ID: **MG983735.1** Length: 8090 Number of Matches: 1
Range 1: 837 to 861

Score	Expect	Identities	Gaps	Strand	Frame
50.1 bits(25)	0.001()	25/25(100%)	0/25(0%)	Plus/Plus	

Features:

```
Query  1    ACAGGCTAAGGATGCCCTTCAGGTA  25
            |||||||||||||||||||||||||
Sbjct  837  ACAGGCTAAGGATGCCCTTCAGGTA  861
```

Foot-and-mouth disease virus - type O isolate SRL/30/2014, partial genome
Sequence ID: **MG983734.1** Length: 8175 Number of Matches: 1
Range 1: 925 to 949

Score	Expect	Identities	Gaps	Strand	Frame
50.1 bits(25)	0.001()	25/25(100%)	0/25(0%)	Plus/Plus	

Features:

```
Query  1    ACAGGCTAAGGATGCCCTTCAGGTA  25
            |||||||||||||||||||||||||
Sbjct  925  ACAGGCTAAGGATGCCCTTCAGGTA  949
```

Foot-and-mouth disease virus - type O isolate SRL/28/2014, partial genome
Sequence ID: **MG983733.1** Length: 8182 Number of Matches: 1
Range 1: 926 to 950

Score	Expect	Identities	Gaps	Strand	Frame
50.1 bits(25)	0.001()	25/25(100%)	0/25(0%)	Plus/Plus	

Features:

```
Query  1    ACAGGCTAAGGATGCCCTTCAGGTA  25
            |||||||||||||||||||||||||
Sbjct  926  ACAGGCTAAGGATGCCCTTCAGGTA  950
```

Foot-and-mouth disease virus - type O isolate SRL/1/2014, partial genome
Sequence ID: **MG983732.1** Length: 8191 Number of Matches: 1
Range 1: 930 to 954

Score	Expect	Identities	Gaps	Strand	Frame
50.1 bits(25)	0.001()	25/25(100%)	0/25(0%)	Plus/Plus	

Features:

```
Query  1    ACAGGCTAAGGATGCCCTTCAGGTA  25
            |||||||||||||||||||||||||
Sbjct  930  ACAGGCTAAGGATGCCCTTCAGGTA  954
```

Foot-and-mouth disease virus - type O isolate SAU/2/2016, complete genome
Sequence ID: **MG983729.1** Length: 8182 Number of Matches: 1
Range 1: 932 to 956

Score	Expect	Identities	Gaps	Strand	Frame
50.1 bits(25)	0.001()	25/25(100%)	0/25(0%)	Plus/Plus	

Features:

```
Query  1    ACAGGCTAAGGATGCCCTTCAGGTA  25
            |||||||||||||||||||||||||
Sbjct  932  ACAGGCTAAGGATGCCCTTCAGGTA  956
```

Foot-and-mouth disease virus - type O isolate SAU/1/2016, partial genome
Sequence ID: **MG983728.1** Length: 8146 Number of Matches: 1
Range 1: 909 to 933

Score	Expect	Identities	Gaps	Strand	Frame
50.1 bits(25)	0.001()	25/25(100%)	0/25(0%)	Plus/Plus	

Features:

```
Query  1    ACAGGCTAAGGATGCCCTTCAGGTA  25
            |||||||||||||||||||||||||
Sbjct  909  ACAGGCTAAGGATGCCCTTCAGGTA  933
```

Foot-and-mouth disease virus - type O isolate SAU/20/2015, partial genome
Sequence ID: **MG983727.1** Length: 8177 Number of Matches: 1
Range 1: 931 to 955

Score	Expect	Identities	Gaps	Strand	Frame
50.1 bits(25)	0.001()	25/25(100%)	0/25(0%)	Plus/Plus	

Features:

```
Query  1    ACAGGCTAAGGATGCCCTTCAGGTA  25
            |||||||||||||||||||||||||
Sbjct  931  ACAGGCTAAGGATGCCCTTCAGGTA  955
```

Foot-and-mouth disease virus - type O isolate SAU/4/2014, partial genome
Sequence ID: **MG983726.1** Length: 8208 Number of Matches: 1
Range 1: 933 to 957

Score	Expect	Identities	Gaps	Strand	Frame
50.1 bits(25)	0.001()	25/25(100%)	0/25(0%)	Plus/Plus	

Features:

```
Query  1    ACAGGCTAAGGATGCCCTTCAGGTA  25
            |||||||||||||||||||||||||
Sbjct  933  ACAGGCTAAGGATGCCCTTCAGGTA  957
```

Foot-and-mouth disease virus - type O isolate SAU/1/2014, partial genome
Sequence ID: **MG983725.1** Length: 8205 Number of Matches: 1
Range 1: 932 to 956

Score	Expect	Identities	Gaps	Strand	Frame
50.1 bits(25)	0.001()	25/25(100%)	0/25(0%)	Plus/Plus	

Features:

```
Query  1    ACAGGCTAAGGATGCCCTTCAGGTA  25
            |||||||||||||||||||||||||
Sbjct  932  ACAGGCTAAGGATGCCCTTCAGGTA  956
```

Foot-and-mouth disease virus - type O isolate SAU/7/2013, partial genome
Sequence ID: **MG983724.1** Length: 8173 Number of Matches: 1
Range 1: 922 to 946

Score	Expect	Identities	Gaps	Strand	Frame
50.1 bits(25)	0.001()	25/25(100%)	0/25(0%)	Plus/Plus	

Features:

```
Query  1    ACAGGCTAAGGATGCCCTTCAGGTA  25
            |||||||||||||||||||||||||
Sbjct  922  ACAGGCTAAGGATGCCCTTCAGGTA  946
```

Foot-and-mouth disease virus - type O isolate SAU/6/2013, partial genome
Sequence ID: **MG983723.1** Length: 8186 Number of Matches: 1
Range 1: 928 to 952

Score	Expect	Identities	Gaps	Strand	Frame
50.1 bits(25)	0.001()	25/25(100%)	0/25(0%)	Plus/Plus	

Features:

```
Query  1    ACAGGCTAAGGATGCCCTTCAGGTA  25
            |||||||||||||||||||||||||
Sbjct  928  ACAGGCTAAGGATGCCCTTCAGGTA  952
```

Foot-and-mouth disease virus - type O isolate SAU/4/2013, partial genome
Sequence ID: **MG983722.1** Length: 8184 Number of Matches: 1
Range 1: 928 to 952

Score	Expect	Identities	Gaps	Strand	Frame
50.1 bits(25)	0.001()	25/25(100%)	0/25(0%)	Plus/Plus	

Features:

```
Query  1    ACAGGCTAAGGATGCCCTTCAGGTA  25
            |||||||||||||||||||||||||
Sbjct  928  ACAGGCTAAGGATGCCCTTCAGGTA  952
```

Foot-and-mouth disease virus - type O isolate SAU/1/2013, partial genome

Sequence ID: **MG983721.1** Length: 8183 Number of Matches: 1

Range 1: 927 to 951

Score	Expect	Identities	Gaps	Strand	Frame
50.1 bits(25)	0.001()	25/25(100%)	0/25(0%)	Plus/Plus	

Features:

```
Query  1    ACAGGCTAAGGATGCCCTTCAGGTA  25
            |||||||||||||||||||||||||
Sbjct  927  ACAGGCTAAGGATGCCCTTCAGGTA  951
```
