# Supplementary material for: Silencing of the foot-and-mouth disease virus internal ribosomal entry site by targeting relatively conserved region among serotypes
Source: Virus Genes. 2019 Jul 31;55(6):786–94. doi: 10.1007/s11262-019-01696-6 (PMC6831537; doi:10.1007/s11262-019-01696-6)
Supplement: Supplementary file 2 — Supplementary material 2 (PDF 13719 kb) Supplementary Fig. 2 Structure of the bicistronic luciferase reporter construct A bicistronic reporter construct was designed to contain the FMDV-IRES element located between the Renilla and firefly luciferase genes. The bicistronic reporter gene was cloned using the restriction enzymes EcoRV and BamHI and was ligated into the pCAGGS-Neo/MCS vector digested with EcoRV and BamHI. Supplementary Fig. 3 Construction of the FMDV-IRES-targeting shRNA expression vector. (A) The FMDV-IRES-targeting shRNA expression vector was constructed using the pLL3.7 vector. CMV: human cytomegalovirus; EGFP: enhanced green fluorescent protein; 5′LTR: truncated 5′ long terminal repeat from HIV-1; RRE: Rev response element of HIV-1; WPRE: woodchuck hepatitis virus posttranscriptional regulatory element. (B) Transfection of cells with shRNA expression vector was observed by fluorescence microscopy (a–f). Fluorescent images (a, g: 40 × ; d: 200 ×), translucent images (b, h: 40 × ; e: 200 ×), and merged images (c, i: 40 × ; f: 200 ×) are shown. Mock transfection controls (g–i) are also shown [file 11262_2019_1696_MOESM2_ESM.pdf]

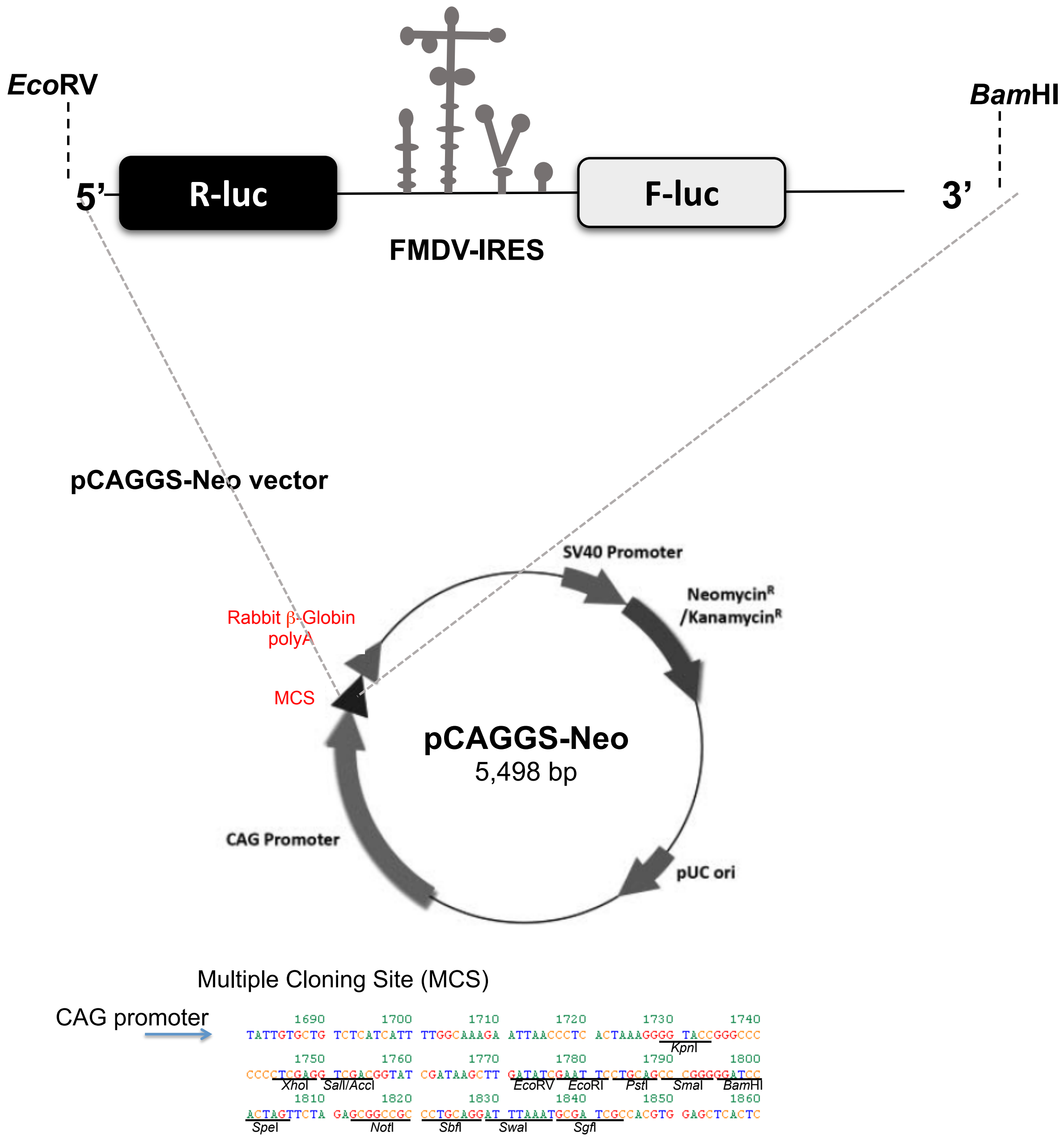

Supplementary Fig.2

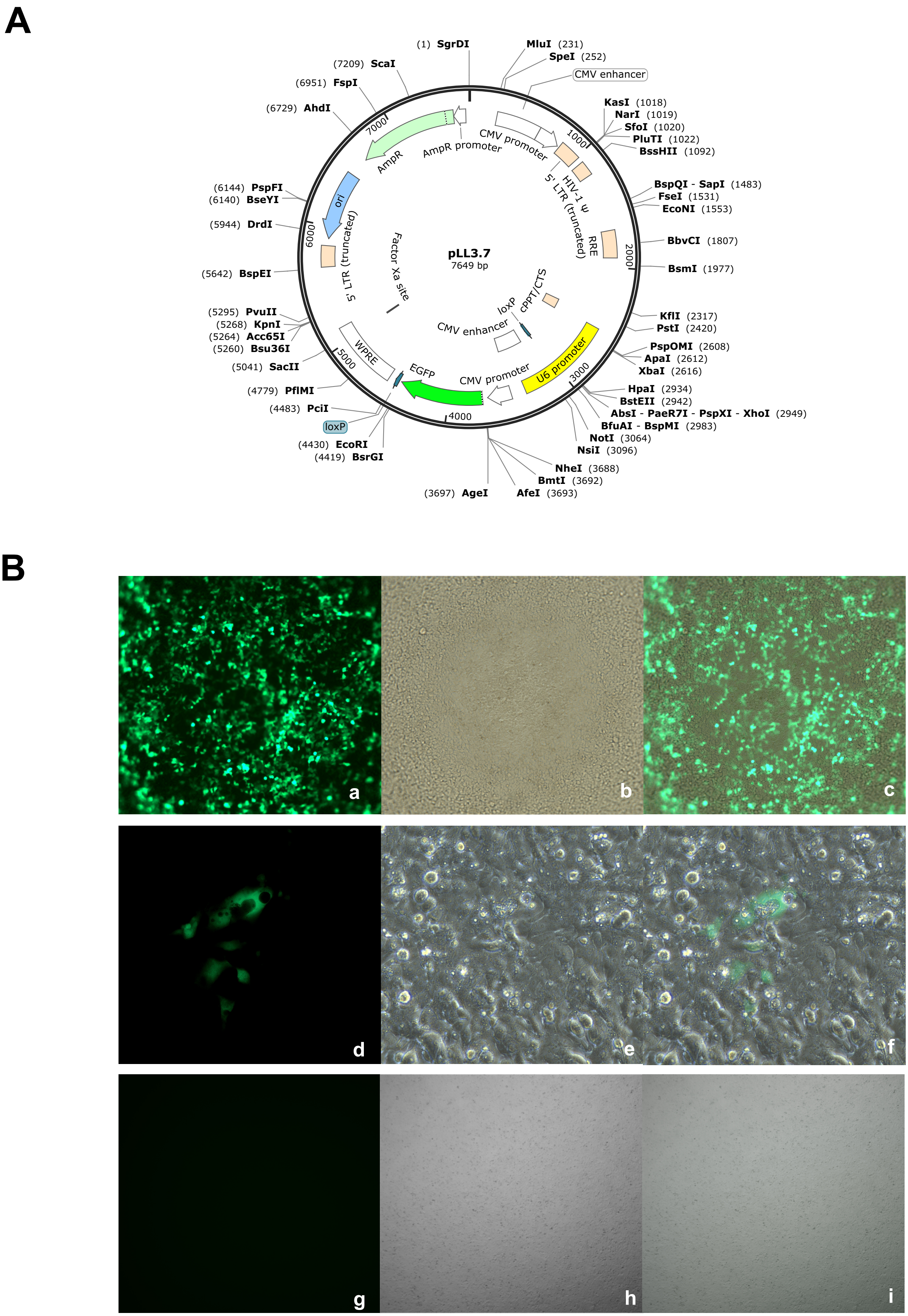

Supplementary Fig.3
